# Supplementary material for: Causal Relationship between Aging and Anorexia Nervosa: A White-Matter-Microstructure-Mediated Mendelian Randomization Analysis
Source: Biomedicines. 2024 Aug 16;12(8):1874. doi: 10.3390/biomedicines12081874 (PMC11351342; doi:10.3390/biomedicines12081874)
Supplement: Supplementary file 1 [file biomedicines-12-01874-s001.zip › Supplementary Figure/Figure S4/Singe SNP plot of MR analysis for TL to ubm-b-1811.pdf]

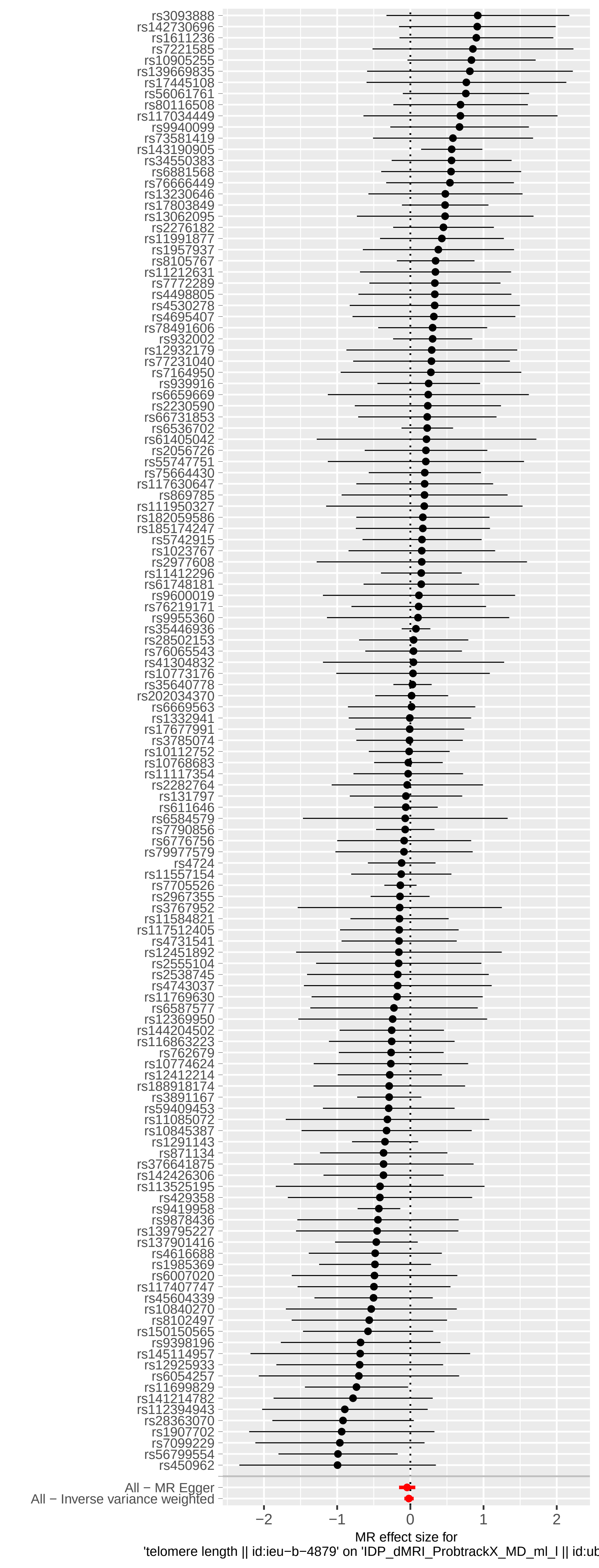
rs3093888
rs142730696
rs1611236
rs7221585
rs10905255
rs139669835
rs17445108
rs56061761
rs80116508
rs117034449
rs9940099
rs73581419
rs143190905
rs34550383
rs6881568
rs76666449
rs13230646
rs17803849
rs13062095
rs2276182
rs11991877
rs1957937
rs8105767
rs11212631
rs7772289
rs4498805
rs4530278
rs4695407
rs78491606
rs932002
rs12932179
rs77231040
rs7164950
rs939916
rs6659669
rs2230590
rs66731853
rs6536702
rs61405042
rs2056726
rs55747751
rs75664430
rs117630647
rs869785
rs111950327
rs182059586
rs185174247
rs5742915
rs1023767
rs2977608
rs11412296
rs61748181
rs9600019
rs76219171
rs9955360
rs35446936
rs28502153
rs76065543
rs41304832
rs10773176
rs35640778
rs202034370
rs6669563
rs1332941
rs17677991
rs3785074
rs10112752
rs10768683
rs11117354
rs2282764
rs131797
rs611646
rs6584579
rs7790856
rs6776756
rs79977579
rs4724
rs11557154
rs7705526
rs2967355
rs3767952
rs11584821
rs117512405
rs4731541
rs12451892
rs2555104
rs2538745
rs4743037
rs11769630
rs6587577
rs12369950
rs144204502
rs116863223
rs762679
rs10774624
rs12412214
rs188918174
rs3891167
rs59409453
rs11085072
rs10845387
rs1291143
rs871134
rs376641875
rs142426306
rs113525195
rs429358
rs9419958
rs9878436
rs139795227
rs137901416
rs4616688
rs1985369
rs6007020
rs117407747
rs45604339
rs10840270
rs8102497
rs150150565
rs9398196
rs145114957
rs12925933
rs6054257
rs11699829
rs141214782
rs112394943
rs28363070
rs1907702
rs7099229
rs56799554
rs450962
All − MR Egger
All − Inverse variance weighted
−2
−1
0
1
2
MR effect size for
'telomere length || id:ieu−b−4879' on 'IDP_dMRI_ProbtrackX_MD_ml_l || id:ub
